# The Peripheral and Intratumoral Immune Cell Landscape in Cancer Patients: A Proxy for Tumor Biology and a Tool for Outcome Prediction

**DOI:** 10.3390/biomedicines6010025

**Published:** 2018-02-24

**Authors:** Annette Schnell, Christian Schmidl, Wolfgang Herr, Peter J. Siska

**Affiliations:** 1 Department of Internal Medicine III, Hematology and Oncology, University Hospital Regensburg, 93053 Regensburg, Germany; annette.schnell@ukr.de (A.S.); wolfgang.herr@ukr.de (W.H.); 2 Regensburg Centre for Interventional Immunology and University Medical Center of Regensburg, 93053 Regensburg, Germany; christian.schmidl@ukr.de

**Keywords:** tumor, tumor-infiltrating, peripheral blood, immune cells, prognosis, prediction, tools

## Abstract

Functional systemic and local immunity is required for effective anti-tumor responses. In addition to an active engagement with cancer cells and tumor stroma, immune cells can be affected and are often found to be dysregulated in cancer patients. The impact of tumors on local and systemic immunity can be assessed using a variety of approaches ranging from low-dimensional analyses that are performed on large patient cohorts to multi-dimensional assays that are technically and logistically challenging and are therefore confined to a limited sample size. Many of these strategies have been established in recent years leading to exciting findings. Not only were analyses of immune cells in tumor patients able to predict the clinical course of the disease and patients’ survival, numerous studies also detected changes in the immune landscape that correlated with responses to novel immunotherapies. This review will provide an overview of established and novel tools for assessing immune cells in tumor patients and will discuss exemplary studies that utilized these techniques to predict patient outcomes.

## 1. Introduction

Described as “wounds that never heal” [1], tumors are intimately associated with inflammation and immune cells that can both promote and suppress cancer growth. Numerous strategies that aim to modulate immune cells and inhibit tumor growth have been tested pre-clinically and clinically. In the last decade, the re-activation of “exhausted” endogenous tumor-reactive immune cells or administration of ex vivo expanded cells gained much attention and numerous patients already benefited from these approaches. In addition to the therapeutic potential, peripheral and tumor-infiltrating immune cells react to local and systemic signals caused by malignant growth, and can, therefore, be used as a proxy for disease course. Indeed, a large number of studies described immune cells in cancer patients as being dysregulated. Investigating immune cell dysregulation is complicated by the variety of methods used, a considerable intratumoral heterogeneity, and the impact of tumor environment. In addition to the heterogeneous nature of tumors, various immune cell populations have the potential to both be affected by and actively engage with tumor cells. Accordingly, cells from the adaptive (T and B cells) and innate immunity (macrophages, dendritic cells, natural killer cells and others) have been studied in cancer patients and in animal models. However, in line with the heterogeneity of “inflammation”, conclusions about the prognostic value of distinct immune processes in cancer are often contradictory. In addition, a considerable proportion of published data describing the biological role of a specific immune population in tumor regression or progression has not been reproduced by independent investigators. 

**Figure 1:**
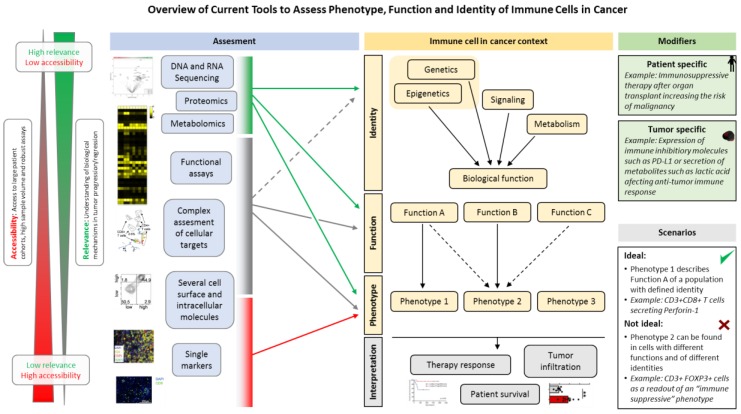
Overview of current tools to assess phenotype, function, and identity of immune cells in cancer. Multiple tools that differ in their accessibility, feasibility and relevance are available to study immune cells that infiltrate human tumors or are found in the periphery. Techniques such as genome or transcriptome sequencing provide deep insights into the identity, function and phenotype of studied targets, they are however technically and logistically challenging and often an inadequate data analysis and interpretation prevent a high information yield. On the contrary, low-dimensional assays (e.g., immunohistochemistry or low-parameter flow cytometry) are broadly accessible, while insufficient to capture the biological complexity. In addition, immune cells in the cancer environment are highly heterogeneous and often no linear relationship between cellular identities, function and phenotype exist due to cellular plasticity and context (e.g., tissue) specificities. Data from [2,3] were used to prepare this figure.

Historically, the knowledge about a specific immune cell population that has been collected in a non-malignant setting, such as in infection, has often been transferred to cancer. Due to the non-malignant nature of immune cells and absence of “micro-evolution” through mutation as known from cancer cells, it is often assumed that the phenotype and biology of immune cells are stable. Therefore, the most widely used approaches in monitoring immune cells in cancer patients involve an assessment of single or low-number of markers at a defined time-point. While highly accessible, technically and logistically feasible, these approaches may fail to describe the biological processes and to identify future therapeutic targets. In contrast, novel tools that aim to assess the complexity of immune cells in cancer are more capable of capturing the heterogeneity and diverse functionality of cells of interest. Indeed, in recent years, the establishment of high-dimensional assays such as numerous–omics (e.g., proteomics, metabolomics, and others) as well as single cell approaches (such as single-cell RNA sequencing) allowed insights of unprecedented depth and of high biological relevance (Figure 1). Here, we will focus on the immune system as a proxy for tumor biology and as a tool to predict or monitor immunotherapy response. Due to the extent of available data, we will mostly discuss studies involving T cells.

## 2. Phenotype and Function of Peripheral Blood Immune Cells as an Outcome Predictor

It has been long known that systemic immunosuppression induced in patients after organ transplant can increase the incidence of cancer. Furthermore, patients with established tumors still profit from a functional immune system [4]. The most widely used source of immune cells from cancer patients is peripheral blood as it is highly accessible and can be processed with high consistency across patients and institutions. As opposed to immune cells infiltrating tumor tissues, blood immune cells can be assessed repeatedly, allowing dynamic measurements during disease progression/regression and under systemic immunotherapy. The accessibility of peripheral blood warrants a high yield of data that can be used to generate biomarkers and stratify patients; however, analyses of blood immune cells may not allow deep insights into the tumor-immune cell interactions. Here we will address how immune populations in patients’ blood can be used to predict survival or immunotherapy response. Exemplary studies will be discussed and the pitfalls of the proposed diagnostic strategies will be considered.

Several investigators correlated changes in peripheral blood immune populations with prognosis of cancer patients. Due to an overwhelming amount of collected data, exemplary studies that focused on T cells will be discussed. T cells develop and differentiate to different subtypes. While CD8+ T cells are mostly pro-inflammatory, CD4+ T cells can be both pro- and anti-inflammatory. As opposed to “effector” CD4+ T cells that are often described as T-helper (Th)-1, Th-2 and Th-17 based on the expression of key T cell cytokines, the immunosuppressive regulatory CD4+ T cells (Treg) are mostly defined by expression of CD25 and the transcription factor forkhead-box-protein P3 (FOXP3). In line with the hypothesis that Treg may inhibit anti-tumor immunity and contribute to cancer progression, increased percentages of Treg were observed in a poor-prognosis subgroup of patients with resectable pancreatic cancer [5]. Similarly, early-stage treatment-naïve non-small cell lung cancer (NSCLC) patients showed an increased percentage of CD4+ FOXP3+ T cells in peripheral blood [6]. Interestingly, NSCLC patients presented lower percentages of cells expressing the immune inhibitory receptor programmed cell death protein 1 (PD-1) as compared to healthy controls. Nevertheless, the PD-1 expression on CD4+ T cells within the NSCLC patient group correlated with poor outcomes in the NSCLC group and a low CD4/CD8 ratio predicted a better prognosis [6]. Ihara et al. analyzed a cohort of head and neck squamous cell carcinoma (HNSCC) patients and found similar percentages of CD4+ FOXP3+ T cells in HNSCC patients as compared to benign tumor patients. Looking at the expression of CD45RA on this T cell subpopulation, however, increased percentages of CD45RA- CD4+ FOXP3+ cells correlated with poor outcomes in HNSCC patients [7]. T follicular helper (Tfh) cells and T follicular regulatory (Tfr) cells are emerging as critical regulators of systemic immunity in various contexts. A recent study assessed circulating Tfh and Tfr in NSCLC patients. Both Tfh and Tfr were increased in NSCLC patients, as compared to healthy controls. In addition, lower percentages of Tfh were found in early stages, but no relationship for Tfr in regard to disease stage was observed. Interestingly, Tfh were preferentially found in patients with a squamous cell carcinoma histology as compared to adenocarcinoma [8]. Immune cells other than T cells may associate with the disease course of tumor patients and have been assessed by a large number of studies so far. For example, solid data are available showing a predictive potential of the neutrophil-to-lymphocyte ratio in several tumor entities [5,9,10,11]. It is however unknown if changes in the neutrophil/lymphocyte cell compartments reflect a specific tumor biology and the biological and translational relevance of these observations remains unclear. 

## 3. The Phenotype-Function Discrepancy and Its Implications for Cancer Immune Monitoring 

A basic phenotype of an immune cell subpopulation provides only limited information about the specific function (Figure 1). For example, human CD4+ T cells express FOXP3 upon activation and FOXP3 expression may not be sufficient to link a Treg phenotype to a suppressive function. This phenomenon may play a role in the prognostic relevance of peripheral blood Treg in cancer patients. While increased Treg correlate with decreased survival of patients with several tumor entities, they can also be associated with improved prognosis. A meta-analysis by Shang et al. that included 17 tumor types showed a survival advantage of colorectal, head and neck and oesophageal cancer patients with increased Treg infiltration [12]. Although no meta-data are available addressing the prognostic role of peripheral blood Treg across multiple tumor types, investigators observed an association between Treg frequencies in peripheral blood and a worse prognosis of patients in some tumor entities [13,14]. Vetsika et al. discriminated peripheral blood Treg of NSCLC patients as naïve, effector and terminal effector Treg. Interestingly, naïve Treg were increased in NSCLC patients and correlated with poor outcome. On the contrary, an increased percentage of terminal effector Treg was associated with an improved survival, suggesting that terminal differentiation may lead to a decreased Treg function and thus to an improved survival [15]. 

Ideally, the suppressive function of these T cells would be consequently assessed to confirm a “Treg identity”. However, this is not yet technically feasible and the mechanisms how Treg may suppress anti-tumor immunity are insufficiently explored. Nevertheless, several studies assessed both phenotype and in vitro function of effector immune cells from tumor patients [16,17,18], but only limited data are available to date in regard to cancer patient prognosis in correlation to peripheral blood immune cell function. One study analyzed the function of peripheral Treg in melanoma patients receiving ipilimumab. Although no significant differences were observed when comparing Treg function before and after 6-week ipilimumab treatment, an increase of Treg suppressive function was associated with poor survival [19]. Another study addressed the relevance of interleukin-17 (IL-17) producing cells in gallbladder cancer patients. Interestingly, IL-17 secreted by a specific T cell subset, the Tɣδ cells, induced tumor vascularization and the presence of IL-17 producing Tɣδ cells was associated with poor patient survival [20].

Although highly biologically relevant, several factors limit the potential of assays measuring the ex vivo function of immune cells from tumor patients. For example, in vitro culture of immune cells is strongly affecting their biology and numerous studies confirmed the differences of in vivo versus in vitro function of specific human immune populations. Therefore, investigators often verify the in vitro results in animal models, link the observed in vitro features to in situ assessments and analyze more than one tumor entity [21,22,23,24,25,26].

## 4. Peripheral Immune Cell Subsets in Tumor Patients Receiving Immunotherapy 

Pharmacological and cellular therapies that aim to induce an anti-tumor immune response have received high attention in recent years. However, many patients are unresponsive or relapse after immunotherapy. It is therefore critical to (1) identify the poor responders in order to adjust the treatment protocols and (2) analyze the resistance mechanisms in order to prevent resistance and improve responses. Peripheral blood offers an easily accessible source of patient immune cells and may be used for immune monitoring und immune therapies. 

Indeed, numerous studies assessed the status and function of peripheral blood immune cells in patients treated with immune therapies such as an immune checkpoint blockade (ICB). Other than in patients with lung cancer where data are limited about peripheral blood immune cells as predictors of immunotherapy response [27,28], investigators repeatedly addressed this question in melanoma, an entity where ICB, especially through a pharmacological blockade of cytotoxic T-lymphocyte-associated protein 4 (CTLA-4), is widely established. Several immune populations have been assessed and correlated with the response to ipilimumab, a CTLA-4 blocking antibody, in melanoma patients. For example, a higher response to ipilimumab has been associated with increased frequencies of peripheral blood CD8+ effector memory T cells [29,30]. Interesting results have been obtained measuring Treg in melanoma patients before and under ipilimumab treatment. High Treg numbers might lead to decreased anti-tumor immunity and could counter-act the activation of the immune system through blockade of the inhibitory receptor CTLA-4. However, Treg express CTLA-4 and might, therefore, be an additional target of CTLA-4 directed therapy. An increased Treg frequency might thus provide high target cell amount and could positively correlate with a response to CTLA-4 blockade. Indeed, Treg frequencies in peripheral blood were associated with better survival and improved response to ipilimumab in melanoma patients [31]. Interestingly, Tarhini et al. observed an increase of Treg in peripheral blood of ipilimumab treated patients, which was associated with improved survival [32]. Specific anti-tumor immunity is critical for tumor rejection and the effects of ICB may be mediated through expansion of tumor-specific T cells. In agreement with this, it has been described that tumor antigen-specific T cells expand in blood of ipilimumab-treated patients [32,33].

## 5. Immune Cells Provide Critical Information about the Tumor Microenvironment

Peripheral blood is technically easy to obtain and process, can be repeatedly accessed over longer time periods and mostly provides a sufficient sample volume. For these reasons, peripheral blood will most likely remain a highly-utilized material for immune population monitoring in tumor patients in the future. While changes in phenotype, function and metabolism of peripheral immune cells may be a critical readout for changes in systemic immunity, they are unlikely representative of changes directly induced by tumors. Since the variability of relevant tumor antigens is high both among tumor entities and among patients with the same tumor type, the identification of tumor-specific cells of the adaptive immunity in peripheral blood will remain highly challenging. Therefore, immune cells that physically reside inside tumors are likely the best source of information about the tumor-immune cell interactions. However, due to advances in cancer diagnostics and improved surgical techniques, tumors are detected at earlier stages and therefore smaller sizes and are often removed using a minimal-invasive approach. All of these factors contribute to technical and logistic challenges in tumor tissue processing and analysis with a broad range of assays. Nevertheless, a wide range of studies across all available tumor types focused on tumor-infiltrating immune cells and provided deep insights in tumor biology and in some cases also allowed prediction of patient’s prognosis and response to therapy.

Tumors form unique environments in terms of cellular composition, tissue architecture and metabolic milieu. All of these features are highly relevant for intratumoral immune cells. Furthermore, these factors can determine the effectivity of anti-tumor immune responses. In addition to expression of immune modulatory molecules such as programmed cell death 1 ligand 1 (PD-L1) that can directly impair activation of tumor-infiltrating T cells, the ability of tumors to use metabolic changes to affect anti-tumor immunity has gained high attention in recent years. As an example, high expression of the glucose transporter Glut1 in renal cell carcinoma (RCC) tumors correlated with decreased infiltration with CD8+ T cells [34], suggesting that tumors may deplete critical nutrients such as glucose to impair immune cell activity. In this regard, tumor metabolic activity may be seen as a barrier to effective immune cell engagement [35], reviewed in [36].

Intratumoral immune cells are often being described by “classical” lineage and differentiation patterns. However, the unique character of the intratumoral environment most likely results in changes in immune cell phenotype, function and metabolism, that are both highly variable and tumor specific. Here we will discuss studies that aimed to describe tumor-infiltrating immune cells using various approaches and highlight those where the intratumoral immune landscape allowed prediction of patients’ prognosis or treatment response.

## 6. Frequency and Phenotype of Tumor-Infiltrating Immune Cells as a Prognosis Predictor 

It was with great enthusiasm when tumor biologists first described immune cells in human tumors. In line with the concept of cancer as a site of chronic inflammation [1], it has often been observed that highly inflamed tumors are biologically and clinically more aggressive [37,38,39] and accordingly, inflammation was found to promote cancer growth [40,41], reviewed in [42,43]. It soon became obvious, that cells of both innate and adaptive immunity can be found in tumors. In addition to cells of the myeloid lineage such as macrophages and dendritic cells, CD3+ T cells became an extensively studied immune cell subtype in solid cancers [44]. Here, increased T cell tumor infiltration correlated with a better prognosis in most studies [45,46]. In a recent study, Denkert and colleagues analyzed tumor biopsies from 3771 breast cancer patients and found that high T cell infiltration predicted a better survival for patients with human epidermal growth factor receptor 2 (HER2)-positive tumors and increased tumor-infiltrating lymphocyte (TIL) concentrations predicted a response to neoadjuvant chemotherapy in all tumors. In contrast, high TIL concentrations were negatively prognostic for the subgroup of patients with luminal-HER2-negative tumors [47]. Similarly, some studies reported about an unfavorable prognosis of breast tumors with high T cell infiltration [48], while others described specific breast cancer subtypes, where CD8+ T cell infiltration associates with an improved survival [49]. In another gynecologic malignancy, CD4+ Treg infiltrating cervix carcinomas predicted a worse survival, while infiltration with CD8+ T cells, as well as with Perforin-1 and Granzyme-B positive cells had no prognostic value [50]. 

Overall, the majority of studies reports improved survival of patients that show high intratumoral CD8+ T cell infiltration [51,52]. However, exceptions to this “dogma” have been repeatedly described for various tumor entities, including gastric cancer [53] and renal cell carcinoma [45,54,55]. Interestingly, CD8+ T cells can promote cancer growth in hepatocellular tumors as a consequence of a metabolic dysregulation [56]. Similarly, melanoma-associated CD8+ T cells induce expression of the immune suppressive factors indoleamine 2,3 dioxygenase, PD-L1 and a Treg recruitment to the tumor microenvironment [57]. As opposed to other genitourinary cancers such as testicular tumors [3], CD8+ T cell infiltration seems to correlate with worse prognosis in RCC [45,54]. RCC CD8+ TIL are highly heterogeneous [2] and accessing the bulk CD8+ T cell population may be an inadequate prognostic tool. We and others have observed an increased differentiation of intratumoral RCC CD8+ TIL towards a memory phenotype [2,58]. Correlating with the percentage of memory T cells among RCC TIL, Hotta et al. observed in a multivariate analysis that an increased percentage of intratumoral memory T cells was an independent negative prognostic factor for patient overall survival [58]. The high infiltration and negative prognostic value of T cells in RCC tumors could partially be explained by high RCC vascularization, leading to a decreased density of tertiary lymphoid structures, resulting in recruitment of CD4+ Treg and presence of polyclonal CD8+ T cells with limited cytotoxicity [59]. In a study by Giraldo et al., CD8+ TIL, as well as increased expression of the inflammatory molecules Perforin-1 and Granzyme-B, predicted a shorter survival of RCC patients. However, the subgroup of CD8+ TIL-high RCC tumors that showed a lower expression of the T cell “exhaustion” markers combined with an increased presence of dendritic cell signatures in peritumoral immune aggregates showed a more favorable prognosis [54]. In line with the metabolic barriers to immune cell engagement in tumors, the localization of infiltrating T cells appears to be relevant for their effective activation. Mei et al. compared 30 studies involving 2988 colorectal cancer (CRC) patients and observed that a generalized tumor inflammation associated with an improved survival. While in the infiltration with CD3+, CD8+, or FOXP3+ cells in the tumor center did not affect survival, CD8+ T cell infiltration in the tumor stroma and at the invasive margin correlated with an increased patients’ survival [60]. In addition to immune cells of adaptive immunity, natural killer (NK) cells can be found in tumors and high NK cell infiltration correlated with improved survival in several tumor entities [61,62,63,64,65]. Interestingly, Gentles et al. analyzed several tumor types and observed favorable outcomes with high NK cell infiltration only in solid tumors, with the exception of glioblastoma. A pan-cancer approach in this study, however, did not find a positive role of NK cell signature in tumors [52].

In summary, an extensive amount of data has been collected on the basic phenotype of tumor-infiltrating immune cells; these parameters may however be insufficient to provide a robust prognostic tool, as the tumor-immune cell interaction is dynamic and highly heterogeneous. More information might be gained if the traditional subpopulation phenotyping is combined with spatial distribution. It may, therefore, be critical not to interpret the extent of the tumoral immune cell infiltration, but rather its “quality”, such as biological identity and function.

## 7. Function and Metabolism of Immune Cells Dictate the Outcome of Tumor-Immune Cell Interactions

Assessment of phenotype and identity of an immune cell, such as through mono- or multi-parametric approaches mentioned above provides useful information about the lineage commitment and about the basic biology. Nevertheless, it is the function and the dynamic biologic behavior of these immune cells that affects cancer growth. A simple but highly relevant readout of the immune cell “fitness” is the ability to proliferate. T cell proliferation guarantees a high T cell pool for the necessary function. With the commitment to a specific function throughout differentiation however, the ability of T cell to proliferate decreases. Nevertheless, confronted with a growing number of tumor cells, a proliferative potential of intratumoral T cells may be necessary for an effective anti-tumor immune response. As mentioned above, several studies detected a negative correlation of CD8+ T cell infiltration in RCC tumors with patient prognosis. A study by Nakano et al. confirmed these data, but also observed an improved prognosis of RCC patients where CD8+ TIL showed a preserved ability to proliferate, compared to patients with non-proliferative CD8+ TIL [55]. 

Another readout of immune cell functionality is the production of effector molecules after appropriate stimulation. In agreement with studies in other tumor entities, Singer et al. observed a decreased T cell cytokine production in RCC tumors [34]. While intratumoral tumor necrosis factor alpha (TNF-α) did not show a prognostic value in melanoma [66], the capacity of tumor-specific T cells to secrete the pro-inflammatory cytokine TNF-α and a TNF-α accumulation in tumor tissue has been associated with good prognosis of colorectal cancer patients [67]. In contrast, accumulation of IL-17, typically produced by CD4+ Th17 cells, correlated with worse prognosis in colorectal tumors [68] and cholangiocarcinoma [69]. In line, the expression of receptors for the anti-inflammatory transforming growth factor beta (TGF-β) in tumor tissues associated with a bad prognosis of breast cancer patients [70].

A key prerequisite for immune cell function is the availability of basic nutrients to fuel the changing metabolic demands but also an intact metabolic machinery is necessary to process these nutrients [71,72]. Several, mostly pre-clinical studies assessed the metabolic fitness of intratumoral immune cells. A metabolic dysfunction of immune cells in cancer has been recently described for some tumor entities [2,73,74,75]. The causes of the metabolic dysfunction of tumor-associated immune cells are not sufficiently explored to date. Moreover, no data are available on the possible prognostic relevance of the metabolic status of immune cells. However, tumors and immune cells are interacting metabolically [36,76,77], and assessment of immune cell metabolism may therefore be an attractive tool to assess both the fitness of the immune system and the biology of tumors to predict patient outcomes and therapy response. 

## 8. Tumoral Immune Composition and Therapy Response

Most of the studies that aimed to predict cancer patients’ outcome using immune cell analyses included patients that were treated with various approaches, including chemotherapy and radiotherapy. It cannot, therefore, be excluded, that a specific immune feature, in fact, predicts a therapy response, rather than describing the pro- or anti-tumor potential of an immune population. Few studies addressed this question and analyses of immune cells in tumor patients were indeed able to predict how a patient responds to subsequent therapy. In breast cancer, infiltration with T cells, especially with CD8+ T cells predicted good sensitivity to chemotherapy [78]. Similarly, T cell infiltration of tumors predicted sensitivity of rectal cancer patients to radiotherapy [79]. Beuselinck et al. analyzed 53 metastatic RCC patients that were treated with sunitinib, a multi-targeted receptor tyrosine kinase inhibitor. Based on a transcriptome analysis, the patients were segregated into four groups. Interestingly, the group that presented the shortest survival and was therefore sunitinib-resistant showed a strong tumoral inflammatory signature and high expression of PD-1, PD-L1 and a sarcomatoid differentiation [80]. Limited data are available about the prognostic role of intratumoral immune cell composition and function for patients treated with immunotherapies. It has been shown however, that treatment with ipilimumab induced an increase in intratumoral CD8+ T cells of a memory phenotype in advanced melanoma patients [32]. 

## 9. Novel Tools to Improve Predictive Power of Immune Cell Assessment in Cancer Patients 

Although highly accessible and technically robust, the widely used low-dimensional assays, such as immunohistochemistry or low-parameter flow cytometry to assess immune cells in tumor patients may not be sufficient to address the heterogeneity of the extratumoral and intratumoral immune landscape (Figure 1). Therefore, multidimensional strategies such as high-parameter flow cytometry, mass cytometry, DNA and RNA sequencing as well as epigenetic approaches have recently been applied to study cancer-associated immune cells. 

### 9.1. Mass Cytometry 

Similar to flow cytometry, mass cytometry uses antibodies to detect antigens expressed by targets cells, but the employment of metal isotopes for antibody labeling and signal detection allows a simultaneous analysis of tens (or, theoretically, hundreds) of parameters. Mass cytometry has been applied to dissect the phenotype of tumor-infiltrating lymphocytes (TIL) in few studies so far [2,81,82,83,84]. Boddupalli et al. assessed TIL of melanoma patients using mass cytometry and observed a decreased cytokine production and a highly heterogeneous expression of immune checkpoints on TIL [66]. Similarly, we observed a high heterogeneity of the intratumoral CD8+ TIL population in RCC tumors. Multi-parametric analysis on single cell level and comparison to resting and activated peripheral blood T cells allowed differentiation of CD8+ TIL into 3 groups and revealed that the most enriched CD8+ TIL group highly expressed PD-1 while exhibiting an effector memory like phenotype [2] that has previously been linked to poor survival of RCC patients [58]. An extensive mass cytometry-based analysis of RCC TIL has been performed by Chevrier et al. This study broadly described the innate and adaptive immune landscape of RCC TIL and also allowed outcome prediction based on patient allocation to one of specific clusters that have been defined by the co-expression of the pre-defined immune parameters [81]. Using a complex but highly relevant approach, Lavin et al. analyzed malignant and non-malignant lung tissue and peripheral blood samples from the same patients with lung adenocarcinoma. Through employment of mass cytometry, the investigators found significantly altered T and NK cell compartments and identified tumor-infiltrating myeloid cells that likely compromised anti-tumor T cell immunity [83]. A recent study included advanced melanoma patients treated with a PD-1 blocking antibody. Using mass cytometry, the authors analyzed peripheral blood samples before and 12 weeks after initiation of an anti-PD-1 therapy. Interestingly, this approach was able to separate anti-PD-1 therapy responders and non-responders and a clear response to immunotherapy was observed in the T cell compartment. In addition, analysis of samples from untreated patients revealed the frequency of CD14+ CD16− HLA-DR^hi^ monocytes to be a strong predictor of progression-free survival and overall survival following anti-PD-1 treatment [85]. 

### 9.2. Assessment of TCR Clonality 

For an antigen-dependent activation, T cells require ligation of the T cell receptor (TCR) through an antigen presenting cell. Presentation of tumor antigens is believed to primarily occur via antigen-presenting cells in lymphoid structures, but tumor cells may also present major histocompatibility complex-I (MHC-I) restricted antigens directly to immune cells. In line, a clonal expansion of tumor-specific T cells is considered necessary for anti-tumor T cell engagement. The vast majority of solid tumors do not induce a monoclonal T cell expansion, which is in line with the observation that solid tumors mostly do not provide a single tumor-specific antigen. Interestingly, a recent study described the phenotype of melanoma CD8+ TIL as typical for tissue resident T cells and observed an extensive clonal heterogeneity of TIL TCR [66]. Similarly, in RCC, colorectal pancreatic and breast cancer, the TIL composition in regard of TCR clonality appears to be also highly heterogeneous [86,87,88] with an exception in ovarian tumors, where the TCR clonality is relatively homogeneous both intratumorally and between patients [89]. Nevertheless, the TCR repertoire of TIL seems to be more narrow than that of peripheral blood T cells from the same patient and specific TCR clones can be found in blood and tumors that are patient-specific and absent in healthy donors [86,87,88,90]. Giraldo et al. analyzed RCC tumors and combined TCR sequencing with multi-parametric flow cytometry. Comparing two groups of patients with inflamed tumors, those with oligoclonal intratumoral T cells showed a superior survival relative to a group with inflamed tumors but polyclonal TIL. Interestingly, TIL in both groups expressed the “exhaustion” markers PD-1 and T cell immunoglobulin and mucin-domain containing-3 (TIM3). 

### 9.3. Transcriptomics 

Even without a single cell resolution capacity being widely accessible to date, sequencing of transcribed RNA represents one of the broadest approaches to assess the identity and functional state of a cellular population. RNA sequencing has been performed on an extensive cohort of patients throughout most of tumor entities. Lacking single cell data, pre-defined immune signatures are necessary to study the intratumoral immune infiltration using the RNA transcriptome of human tumors. Nevertheless, gene expression profiling in combination with immunohistochemistry was able to provide a more accurate prediction of outcome than a traditional histological approach in colorectal tumors [91]. Another study used gene transcription data to define immune cell infiltration of head and neck squamous cell carcinoma tumors and detected a positive correlation of infiltration with NK cells, CD8+ T cells, but also with Treg and other immune populations with overall survival [92]. Similarly, Gentles et al. applied a massive approach and analyzed the transcriptome data across 39 tumor entities and described shared prognostic features of immune infiltrate signatures that have been defined using the CIBERSORT computational approach [93]. Methods such as CIBERSORT are able to delineate cell composition of bulk samples. However, the bulk data can be deconvoluted only to a certain level. Using expression signatures that have been pre-defined using knowledge of specific populations may only provide read-outs that are fitting the hypotheses, rather than being truly exploratory. The only solution to this experimental “bias” is the assessment of the transcriptome on a single cell level. While already available, this approach suffers from low resolution, high reagent costs and is technically challenging both in data collection and analysis. Recently, several investigators have interrogated the immune cell landscape of primary human cancers, identifying heterogeneous subpopulations of cancerous cells and infiltrating immune cells at the same time [94,95,96].

### 9.4. Epigenomics 

The use of the transcriptome allows a broad assessment of the cellular state in a certain moment. However, true cell identity, potential and stability is better reflected by a cell’s epigenome. Epigenetic mechanisms ensure “imprinting” of a certain cell state that may remain stable despite acute changes in the microenvironment, and thus better reflect the potential of a cell to respond to treatment or define cellular phenotypes for biomarker development. For example, the expression of the transcription factor FOXP3 is commonly used alone or in combination with other markers to define an immunosuppressive phenotype of CD4+ T cells. However, as opposed to murine T cells, FOXP3 is expressed in human T cells that are both immunosuppressive and inflammatory [97,98]. Epigenetic analysis of the Treg-specific demethylated region (TSDR) reveals a specific epigenetic state that is unique to immunosuppressive naïve Treg cells [99]. This phenomenon is very likely also to be found in other traditional markers of cellular identity. Therefore, epigenetic features such as DNA methylation, chromatin accessibility and histone modifications may re-define cellular populations that are established to date. The emerging possibility to assess the epigenome on low cell numbers will allow deep analyses of the cellular state and identity of TIL in the future. Although technically challenging, new methodological developments such as ATAC-seq [100] or ChIPmentation [101] increase the accessibility of the epigenome as a data source by lowering input requirements and simplifying experimental procedures. As an example, first studies started to interrogate the chromatin accessibility landscape of TILs in mouse tumor models and/or primary melanoma samples [102,103], paving the way to connect chromatin signatures in T cells to immunotherapy treatment in the future. Similar to transcriptomics, epigenomic assays have been and will be further developed to single-cell resolution. As examples, single cell assays for DNA methylation sequencing [104,105], chromatin accessibility mapping [106,107,108], and chromatin immunoprecipitation [109] have been published. These approaches have a great potential to describe the cell states of heterogeneous tumor-infiltrating immune cells in an unbiased manner and might therefore contribute to outcome prediction in cancer patients in the future.

### 9.5. Multi-Layer Single Cell Data 

Unfortunately, due to the mentioned technical and organizational challenges, studies that deeply assessed the immune cell infiltrate of human tumors were not yet able to provide a prognostic tool that would routinely enable to either predict outcome or therapy response. However, through a multi-parametric approach, they are likely to discover novel targets of prognostic relevance that can be assessed with broadly accessible technologies. To this end, methods that provide more than one sort of data from a single cell have high potential to refine predictions to treatment in by an unbiased assessment the patient’s immune cell status. First studies used single-cell expression data to assemble TCR sequences of TIL in liver cancer, which gave unprecedented insights into phenotypes and relations of infiltrating CD4+ and CD8+ T cell subsets [94]. Further, by adding spatial information to single-cell RNA-profiles important information about the location and interacting cells of the immune infiltrate can potentially be gained [110]. By labeling antibodies with DNA barcodes, researchers could identify dozens of surface protein markers along the transcriptome in single blood cells [111,112], an approach that is highly scalable and therefore a potential alternative to mass cytometry. In addition to those multi-layer-data-generating approaches that were already applied to immune cells, studies described the simultaneous analysis of the transcriptome and DNA methylome [113], DNA methylation and chromatin accessibility [114], selected proteins and transcripts [115], or even transcriptomes, genomes and DNA methylomes [116] from the same single cell.

### 9.6. Others 

Novel tools based on proteomics, metabolomics and microbiomics allow to broadly assess the landscape of a specific population or anatomical site. Multiple studies that analyzed the proteome and lipidome in sera of tumor patients showed promising results in regard to assessment of therapy response and prognosis [117,118,119,120,121,122]. However, these techniques are highly challenging due to dynamic range of substances, especially when applied to different compartments and tissues. Furthermore, these studies suffer from a low reproducibility due to heterogeneity of the cancer itself, as well as due to the different techniques utilized for protein identification/quantification [123]. 

The human microbiome is intimately connected with several compartments, most prominently with the immune system [124]. An intriguing observation has been made by analyzing the intestinal microbiome of cancer patients. For example, it has been shown that the efficacy of CTLA-4 blockade depends on distinct bacterial species, specifically *Bacteriodes fragilis* [125]. Moreover, melanoma patients treated with CTLA-4 blockade showed improved survival, if their baseline microbiota were enriched with *Faecalibacterium* genus and other *Firmicutes*. On the opposite, patients whose microbiota were driven by *Bacteroides* showed inferior survival under CTLA-4 blockade [126]. Similarly, a successful treatment with PD-1 based immunotherapy was associated with *Akkermansia muciniphila* and oral supplementation with this organism was able to restore responsiveness to PD-1 blockade [127]. The exact mechanisms, how microbiota both react to immune perturbations and affect the immune system remain insufficiently explored. However, these exciting data highlight the complexity of the immune-related changes in tumor patients and also identify novel targets that might be used as future predictive biomarkers. 

## 10. Conclusions

A broad landscape of available technologies that are used to assess immune cells in tumor patients has led to generation of considerable amounts of data across multiple tumor types. Nevertheless, the used approaches differ substantially in the technical and logistical feasibility and analytic depth. The high availability of low-dimensional approaches has allowed for the screening of many patient samples in recent years. However, insufficient description of the cellular phenotype and function, together with high heterogeneity of tumors, led to simplifications and a specific function was often assigned to a simple phenotype. Moreover, several immune cell phenotypes have been established and extensively studied in animal models but were not critically and repeatedly evaluated in human systems. This approach might have contributed to the contradictory results of many human cancer studies and although technically straightforward, an immune phenotyping of peripheral blood or tumor tissue is still not used as a prognostic tool in daily clinical practice. Complex tools such as multi-dimensional cytometry, metabolic assays or approaches based on RNA sequencing are shedding more light on the function of immune cells in cancer. Through technical progress, these technologies may become more accessible in the near future and provide solid tools that will both predict disease outcomes and stratify patients for effective therapies.
